# Supplementary material for: Moving beyond neurons: the role of cell type-specific gene regulation in Parkinson’s disease heritability
Source: NPJ Parkinsons Dis. 2019 Apr 17;5:6. doi: 10.1038/s41531-019-0076-6 (PMC6470136; doi:10.1038/s41531-019-0076-6)
Supplement: Supplementary file 1 — Supplementary Figures & Text. [file 41531_2019_76_MOESM1_ESM.pdf]

Supplementary Notes

Supplementary note 1. URLs

Barres immunopanning, http://www.brainrnaseq.org/; Baseline LDSC annotations, https://data.broadinstitute.org/alkesgroup/LDSCORE/; Exome Aggregation Consoritum, http://exac.broadinstitute.org/; Expression-weighted cell-type enrichment (EWCE), https://github.com/NathanSkene/EWCE; Finucane GTEx annotations, https://data.broadinstitute.org/alkesgroup/LDSCORE/LDSC_SEG_ldscores/; GTEx Portal, https://www.gtexportal.org/; GTEx co-expression modules, https://snca.atica.um.es/coexp/Run/Catalog/; Intervene, https://asntech.shinyapps.io/intervene/; LDSC, https://github.com/bulik/ldsc/wiki; Linnarsson single-cell RNA-sequencing, http://mousebrain.org/; MAGMA, https://ctg.cncr.nl/software/magma; MitoCarta, https://www.broadinstitute.org/scientific-community/science/programs/metabolic-disease-program/publications/mitocarta/mitocarta-in-0; SCZ GWAS summary statistics, https://www.med.unc.edu/pgc/results-and-downloads; The Human Lysosome Gene Database, http://lysosome.unipg.it/; WGCNA, https://labs.genetics.ucla.edu/horvath/CoexpressionNetwork/Rpackages/WGCNA/; WGCNA hierarchical clustering with k-means, https://github.com/juanbot/km2gcn

Supplementary note 2. Consortia acknowledgments

IPDGC ACKNOWLEDGMENTS

We would like to thank all the subjects who donated their time and biological samples to be a part of this study. We also would like to thank all members of the International Parkinson Disease Genomics Consortium (IPDGC). See for a complete overview of members, acknowledgements and funding http://pdgenetics.org/partners.This work was supported in part by the Intramural Research Programs of the National Institute of Neurological Disorders and Stroke (NINDS), the National Institute on Aging (NIA), and the National Institute of Environmental Health Sciences both part of the National Institutes of Health, Department of Health and Human Services; project numbers 1ZIA-NS003154, Z01-AG000949-02 and Z01-ES101986. In addition this work was supported by the Department of Defense (award W81XWH-09-2-0128), and The Michael J Fox Foundation for Parkinson’s Research. This work was supported by National Institutes of Health grants R01NS037167, R01CA141668, P50NS071674, American Parkinson Disease Association (APDA); Barnes Jewish Hospital Foundation; Greater St Louis Chapter of the APDA. The KORA (Cooperative Research in the Region of Augsburg) research platform was started and financed by the Forschungszentrum für Umwelt und Gesundheit, which is funded by the German Federal Ministry of Education, Science, Research, and Technology and by the State of Bavaria. This study was also funded by the German Federal Ministry of Education and Research (BMBF) under the funding code 031A430A, the EU Joint Programme - Neurodegenerative Diseases Research (JPND) project under the aegis of JPND -www.jpnd.eu- through Germany, BMBF, funding code 01ED1406 and iMed - the Helmholtz Initiative on Personalized Medicine. This study is funded by the German National Foundation grant (DFG SH599/6-1) (grant to M.S), Michael J Fox Foundation, and MSA Coalition, USA (to M.S). The French GWAS work was supported by the French National Agency of Research (ANR-08-MNP-012). This study was also funded by France-Parkinson Association, Fondation de France, the French program “Investissements d’avenir” funding (ANR-10-IAIHU-06) and a grant from Assistance Publique-Hôpitaux de Paris (PHRC, AOR-08010) for the French clinical data. This study was also sponsored by the Landspitali University Hospital Research Fund (grant to SSv); Icelandic Research Council (grant to SSv); and European Community Framework Programme 7, People Programme, and IAPP on novel genetic and phenotypic markers of Parkinson’s disease and Essential Tremor (MarkMD), contract number PIAP-GA-2008-230596 MarkMD (to HP and JHu). Institutional research funding IUT20-46 was received of the Estonian Ministry of Education and Research (SK). The McGill study was funded by the Michael J. Fox Foundation and the Canadian Consortium on Neurodegeneration in Aging (CCNA). This study utilized the high-performance computational capabilities

of the Biowulf Linux cluster at the National Institutes of Health, Bethesda, Md. (http://biowulf.nih.gov), and DNA panels, samples, and clinical data from the National Institute of Neurological Disorders and Stroke Human Genetics Resource Center DNA and Cell Line Repository. People who contributed samples are acknowledged in descriptions of every panel on the repository website. We thank the French Parkinson's Disease Genetics Study Group and the Drug Interaction with genes (DIGPD) study group: Y Agid, M Anheim, F Artaud, A-M Bonnet, C Bonnet, F Bourdain, J-P Brandel, C Brefel-Courbon, M Borg, A Brice, E Broussolle, F Cormier-Dequaire, J-C Corvol, P Damier, B Debilly, B Degos, P Derkinderen, A Destée, A Dürr, F Durif, A Elbaz, D Grabli, A Hartmann, S Klebe, P. Krack, J Kraemmer, S Leder, S Lesage, R Levy, E Lohmann, L Lacomblez, G Mangone, L-L Mariani, A-R Marques, M Martinez, V Mesnage, J Muellner, F Ory-Magne, F Pico, V Planté-Bordeneuve, P Pollak, O Rascol, K Tahiri, F Tison, C Tranchant, E Roze, M Tir, M Vérin, F Viallet, M Vidailhet, A You. We also thank the members of the French 3C Consortium: A Alpérovitch, C Berr, C Tzourio, and P Amouyel for allowing us to use part of the 3C cohort, and D Zelenika for support in generating the genome-wide molecular data. We thank P Tienari (Molecular Neurology Programme, Biomedicum, University of Helsinki), T Peuralinna (Department of Neurology, Helsinki University Central Hospital), L Myllykangas (Folkhalsan Institute of Genetics and Department of Pathology, University of Helsinki), and R Sulkava (Department of Public Health and General Practice Division of Geriatrics, University of Eastern Finland) for the Finnish controls (Vantaa85+ GWAS data). We used genome-wide association data generated by the Wellcome Trust Case-Control Consortium 2 (WTCCC2) from UK patients with Parkinson's disease and UK control individuals from the 1958 Birth Cohort and National Blood Service. Genotyping of UK replication cases on ImmunoChip was part of the WTCCC2 project, which was funded by the Wellcome Trust (083948/Z/07/Z). UK population control data was made available through WTCCC1. This study was supported by the Medical Research Council and Wellcome Trust disease centre (grant WT089698/Z/09/Z to NW, JHa, and ASc). As with previous IPDGC efforts, this study makes use of data generated by the Wellcome Trust Case-Control Consortium. A full list of the investigators who contributed to the generation of the data is available from www.wtccc.org.uk. Funding for the project was provided by the Wellcome Trust under award 076113, 085475 and 090355. This study was also supported by Parkinson's UK (grants 8047 and J-0804) and the Medical Research Council (G0700943 and G1100643). Sequencing and genotyping done in McGill University was supported by grants from the Michael J. Fox Foundation, the Canadian Consortium on Neurodegeneration in Aging (CCNA) and in part thanks to funding from the Canada First Research Excellence Fund (CFREF), awarded to McGill University for the Healthy Brains for Healthy Lives (HBHL) program. We thank Jeffrey Barrett and Jason Downing (Illumina Inc) for assistance with the design of the ImmunoChip and NeuroX arrays. DNA extraction work that was done in the UK was undertaken at University College London Hospitals, University College London, who received a proportion of funding from the Department of Health's National Institute for Health Research Biomedical Research Centres funding. This study was supported in part by the Wellcome Trust/Medical Research Council Joint Call in Neurodegeneration award (WT089698) to the Parkinson's Disease Consortium (UKPDC), whose members are from the UCL Institute of Neurology, University of Sheffield, and the Medical Research Council Protein Phosphorylation Unit at the University of Dundee. We thank the Quebec Parkinson's Network (http://rpq-qpn.org) and its members. This work was supported by the Medical Research Council grant MR/N026004/1. The Braineac project was supported by the MRC through the MRC Sudden Death Brain Bank Grant (MR/G0901254) to J.H. P.A.L. was supported by the MRC (grants MR/N026004/1 and MR/L010933/1) and Michael J. Fox Foundation for Parkinson's Research. Mike A. Nalls' participation is supported by a consulting contract between Data Tecnica International and the National Institute on Aging, NIH, Bethesda, MD, USA, as a possible conflict of interest Dr. Nalls also consults for Illumina Inc, Lysosomal Therapeutics Inc, the Michael J. Fox Foundation and Vivid Genomics among others.

SGPD ACKNOWLEDGEMENTS

The SGPD's contribution was supported by the Australian Research Council (ARC) (DP160102400) and the Australian National Health and Medical Research Council (NHMRC) (1078037,1078901, 1103418, 1107258,

1127440, 1113400). Support also came from ForeFront, a large collaborative research group dedicated to the study of neurodegenerative diseases and funded by the NHMRC (Program Grant 1132524, Dementia Research Team Grant 1095127, NeuroSleep Centre of Research Excellence 1060992) and ARC (Centre of Excellence in Cognition and its Disorders Memory Program CE10001021). Simon Lewis was supported by an NHMRC-ARC Dementia Fellowship (1110414) and Glenda Halliday was supported by an NHMRC Fellowship (1079679). The Queensland Parkinson's Project (QPP) was supported by a grant from the Australian National Health and Medical Research Council (1084560) to George Mellick. The New Zealand Brain Research Institute (NZBRI) cohort was funded by a University of Otago Research Grant, together with financial support from the Jim and Mary Carney Charitable Trust (Whangarei, New Zealand).

Supplementary Figures

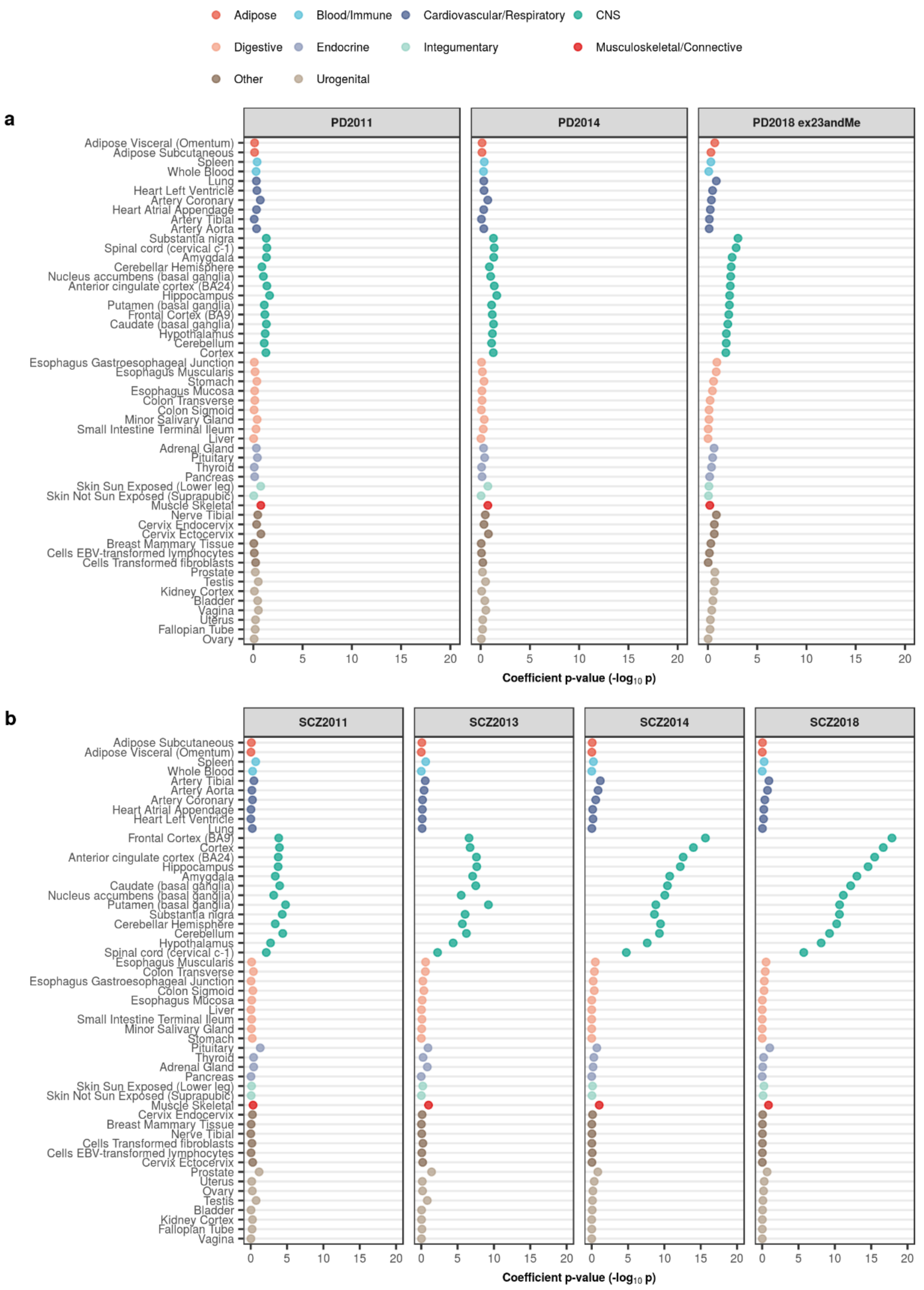

Supplementary Figure 1. Enrichment of PD and SCZ heritability across GWAS iterations in tissue-specific gene expression annotations as used in Finucane *et al.*

(**a**) PD. (**b**) SCZ. Tissues were ordered within each tissue category by the coefficient p-value obtained for (**a**) PD2018 or (**b**) SCZ2018. Numerical results are reported in Supplementary Table 1.

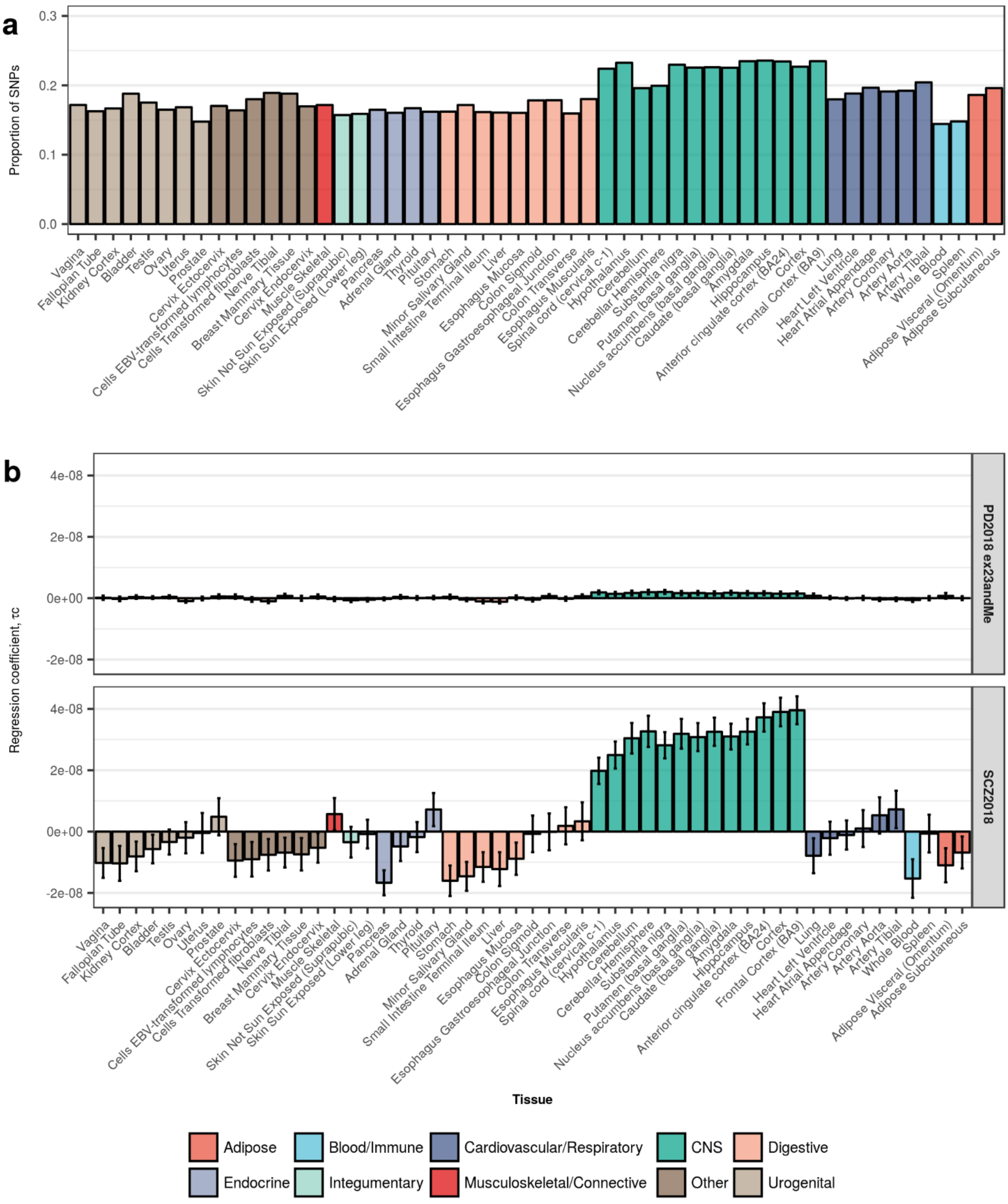

Supplementary Figure 2. SNP proportions (a) and regression coefficients (b) across tissue-specific gene expression annotations. SNP proportions (**a**) are in comparison to the baseline model, consisting of 9,997,231 SNPs. Tissues in (**a, b**) were ordered within each tissue category by the coefficient p-value obtained for SCZ2018. Error bars represent the standard error of the coefficient. This is outputted by the LDSC software, which estimates it using the covariance matrix for coefficient estimates.

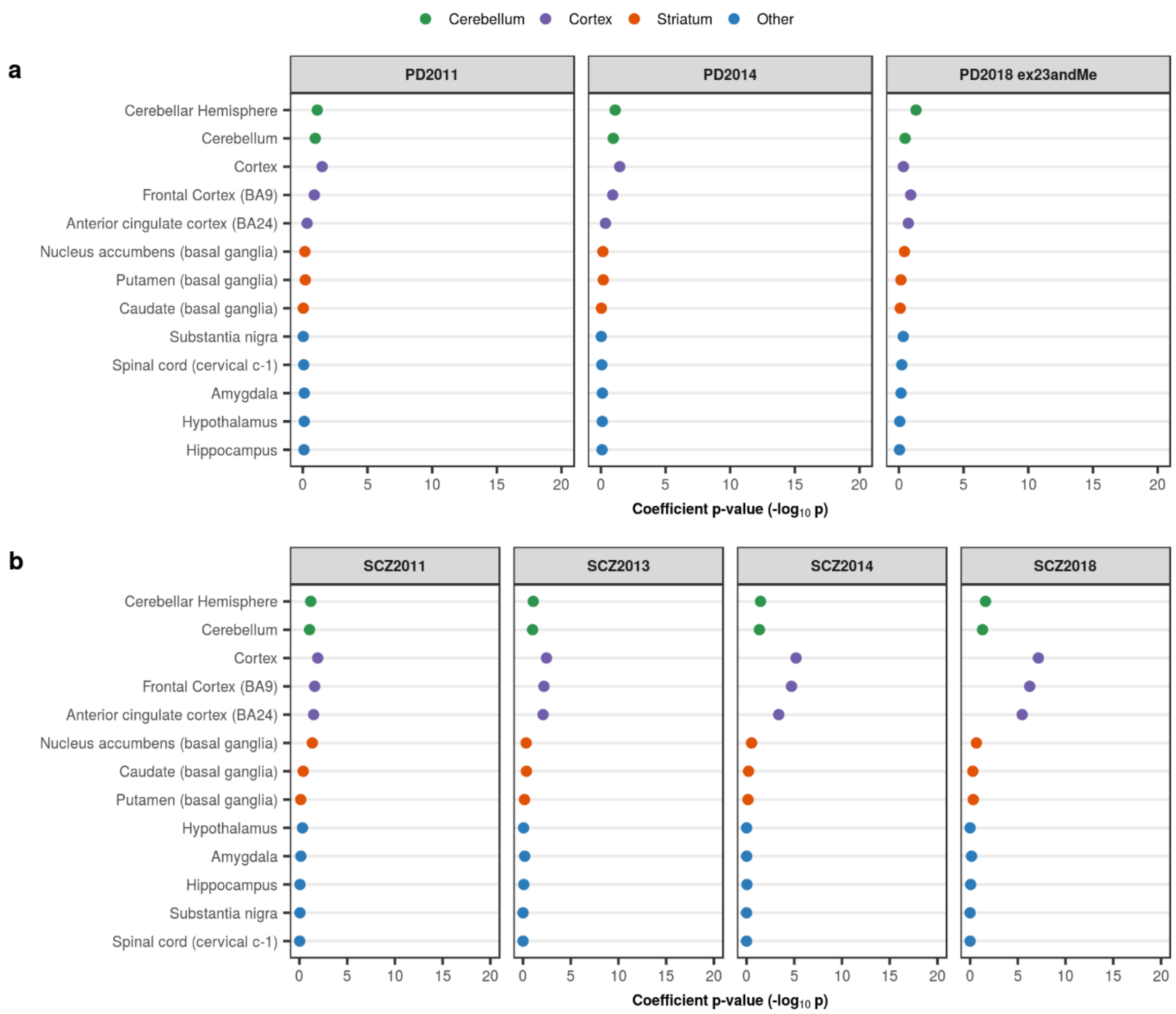

Supplementary Figure 3. Enrichment of PD and SCZ heritability across GWAS iterations in brain-region-specific gene expression annotations as used in Finucane *et al.*
(**a**) PD. (**b**) SCZ. Tissues were ordered within each tissue category by the coefficient p-value obtained for (**a**) PD2018 or (**b**) SCZ2018. Numerical results are reported in Supplementary Table 1.

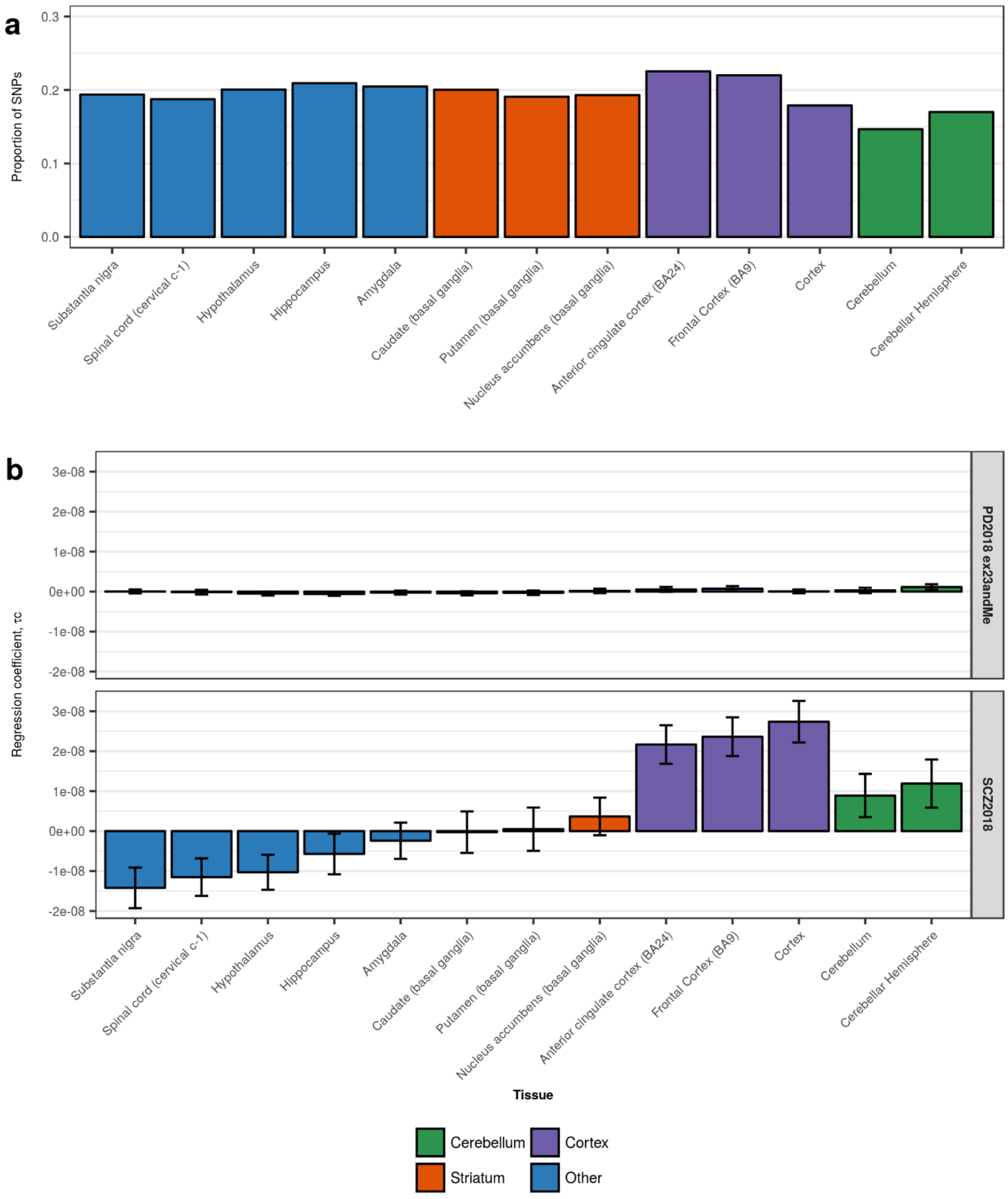

Supplementary Figure 4. SNP proportions (a) and regression coefficients (b) across brain-region-specific gene expression annotations. SNP proportions (**a**) are in comparison to the baseline model, consisting of 9,997,231 SNPs. Tissues in (**a, b**) were ordered within each tissue category by the coefficient p-value obtained for SCZ2018. Error bars represent the standard error of the coefficient. This is outputted by the LDSC software, which estimates it using the covariance matrix for coefficient estimates.

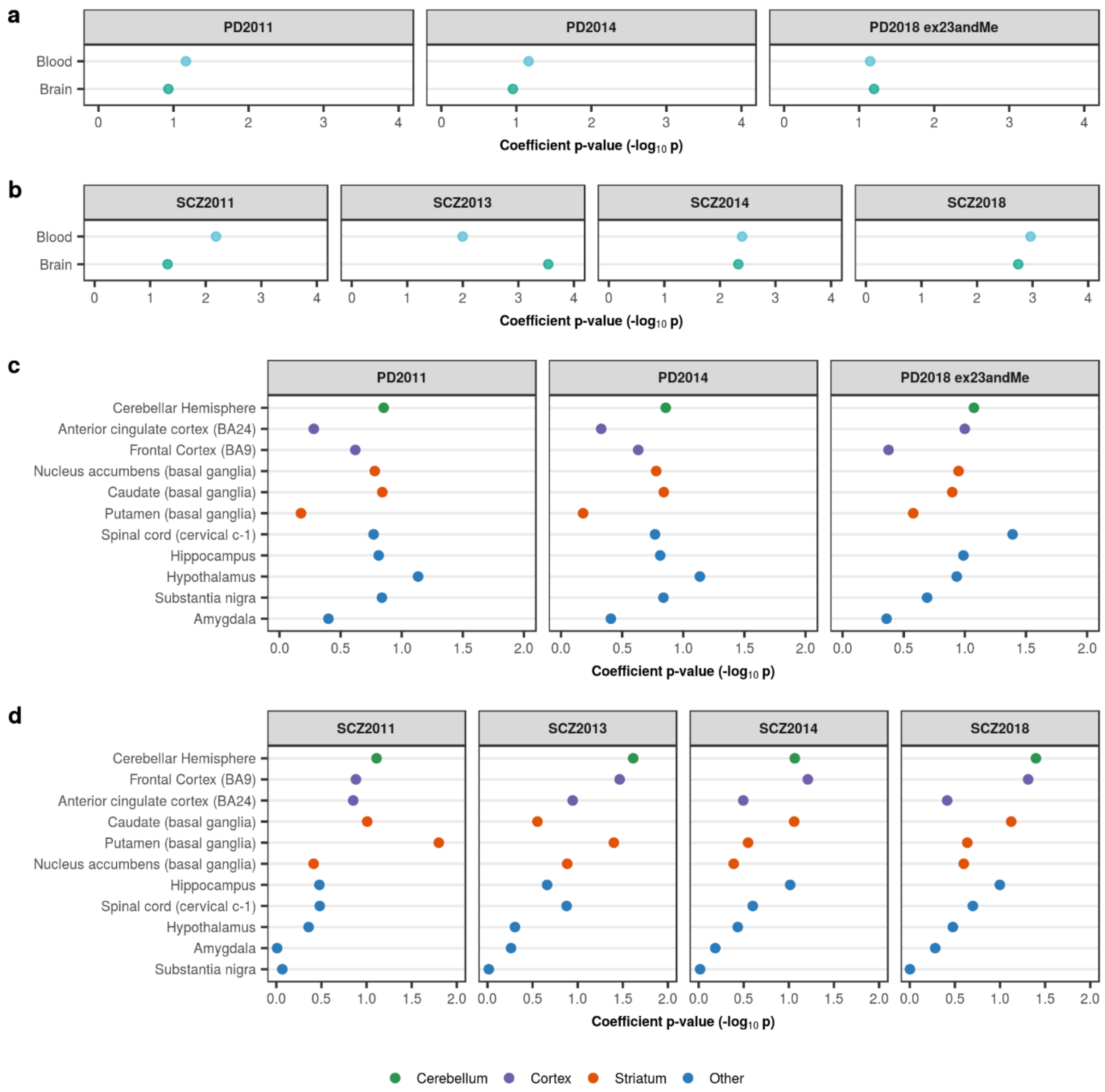

Supplementary Figure 5. Enrichment of PD and SCZ common-SNP heritability across GWAS iterations in tissue-specific eQTL annotations.

Comparison of all brain and blood eQTLs in PD (**a**) and SCZ (**b**). Within-brain comparison of region-specific eQTLs in PD (**c**) and SCZ (**d**). In (**c, d**)Tissues were ordered within each tissue category by the coefficient p-value obtained for (**c**) PD2018 or (**d**) SCZ2018. Numerical results are reported in Supplementary Table 2.

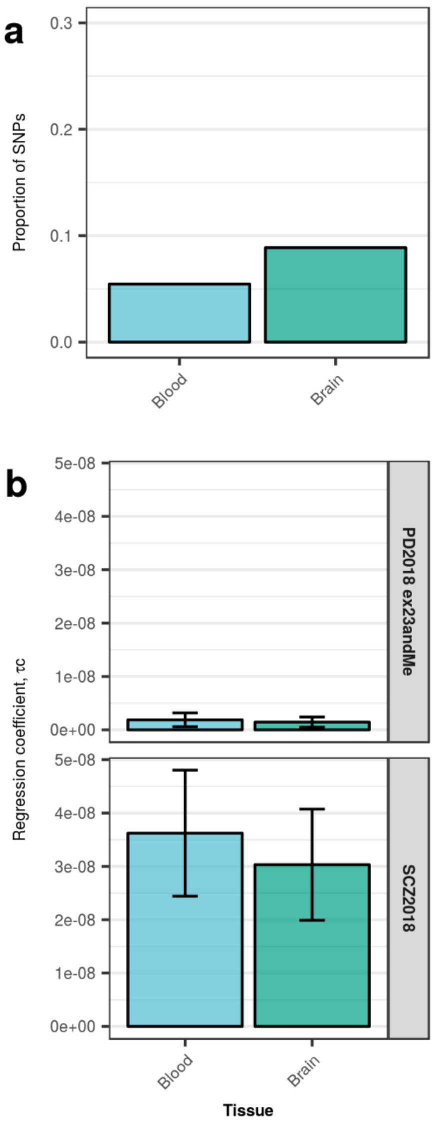

Supplementary Figure 6. SNP proportions (a) and regression coefficients (b) across blood and brain-specific eQTL annotations. SNP proportions (**a**) are in comparison to the baseline model, consisting of 9,997,231 SNPs. Tissues in (**a, b**) were ordered alphabetically. Error bars represent the standard error of the coefficient. This is outputted by the LDSC software, which estimates it using the covariance matrix for coefficient estimates.

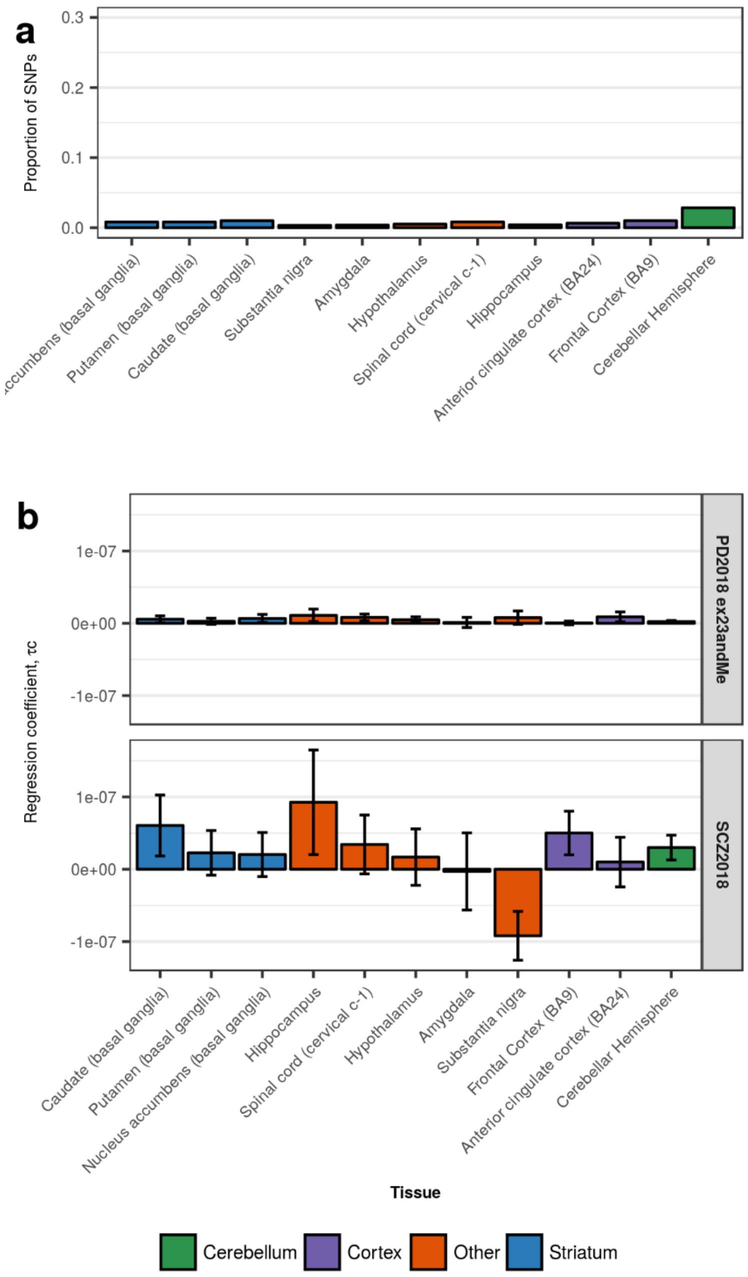

Supplementary Figure 7. SNP proportions (a) and regression coefficients (b) across brain-region-specific eQTL annotations. SNP proportions (**a**) are in comparison to the baseline model, consisting of 9,997,231 SNPs. Tissues (**a, b**) were ordered within each tissue category by the coefficient p-value obtained for SCZ2018. Error bars represent the standard error of the coefficient. This is outputted by the LDSC software, which estimates it using the covariance matrix for coefficient estimates.

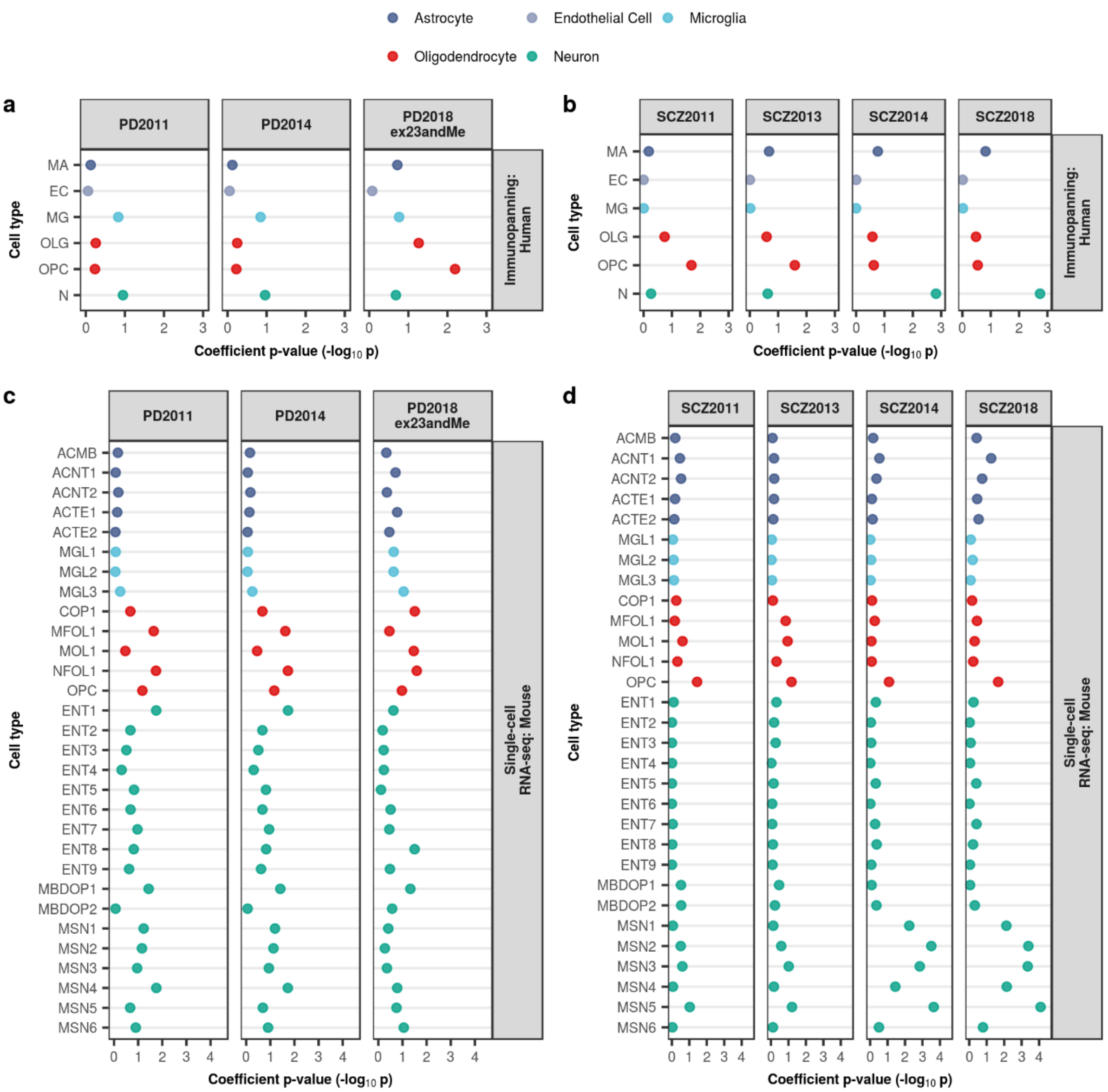

Supplementary Figure 8. Enrichment of PD and SCZ common-SNP heritability across GWAS iterations in brain-related cell-type-specific gene expression annotations.

Results for PD (**a**) and SCZ (**b**) in immunopanned cell types from human temporal lobe. Results for PD (**c**) and SCZ (**d**) in single-cell RNA-sequencing of the adolescent mouse nervous system. Cell types were ordered alphabetically within each overarching cell type category. Numerical results and cell-type abbreviations are reported in Supplementary Table 3.

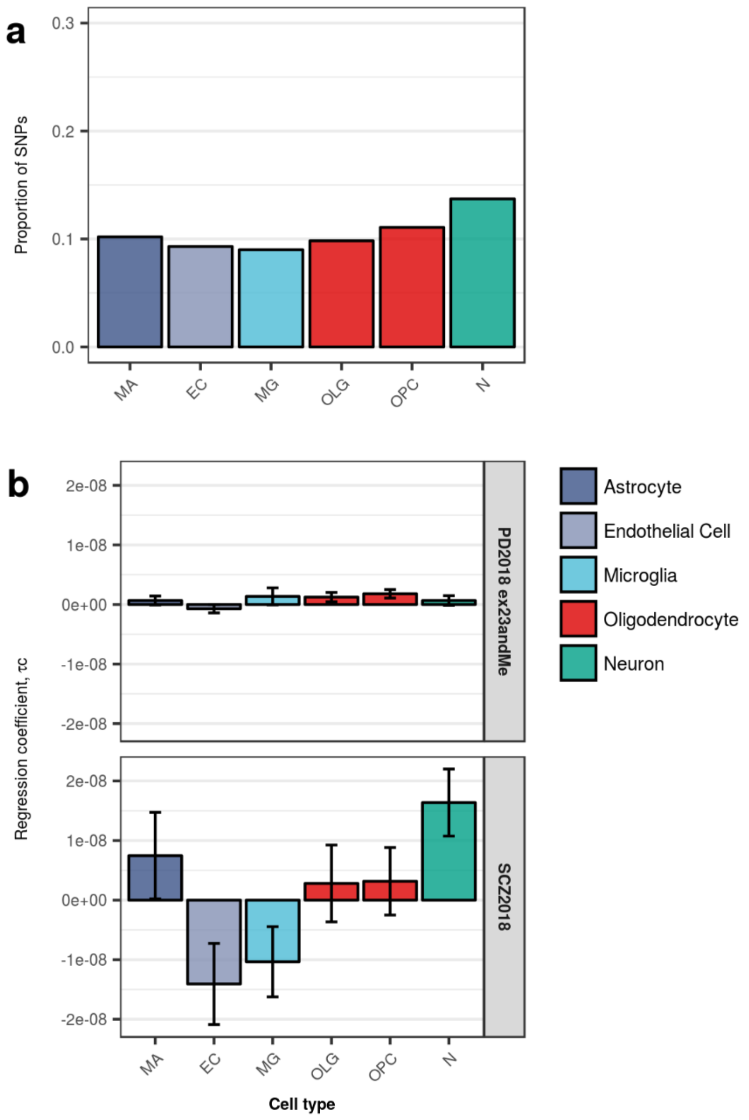

Supplementary Figure 9. SNP proportions (a) and regression coefficients (b) across immunopanning-based cell-type-specific gene expression annotations. SNP proportions (**a**) are in comparison to the baseline model, consisting of 9,997,231 SNPs. Cell types (**a, b**) were ordered alphabetically within each overarching cell type category. Error bars represent the standard error of the coefficient. This is outputted by the LDSC software, which estimates it using the covariance matrix for coefficient estimates.

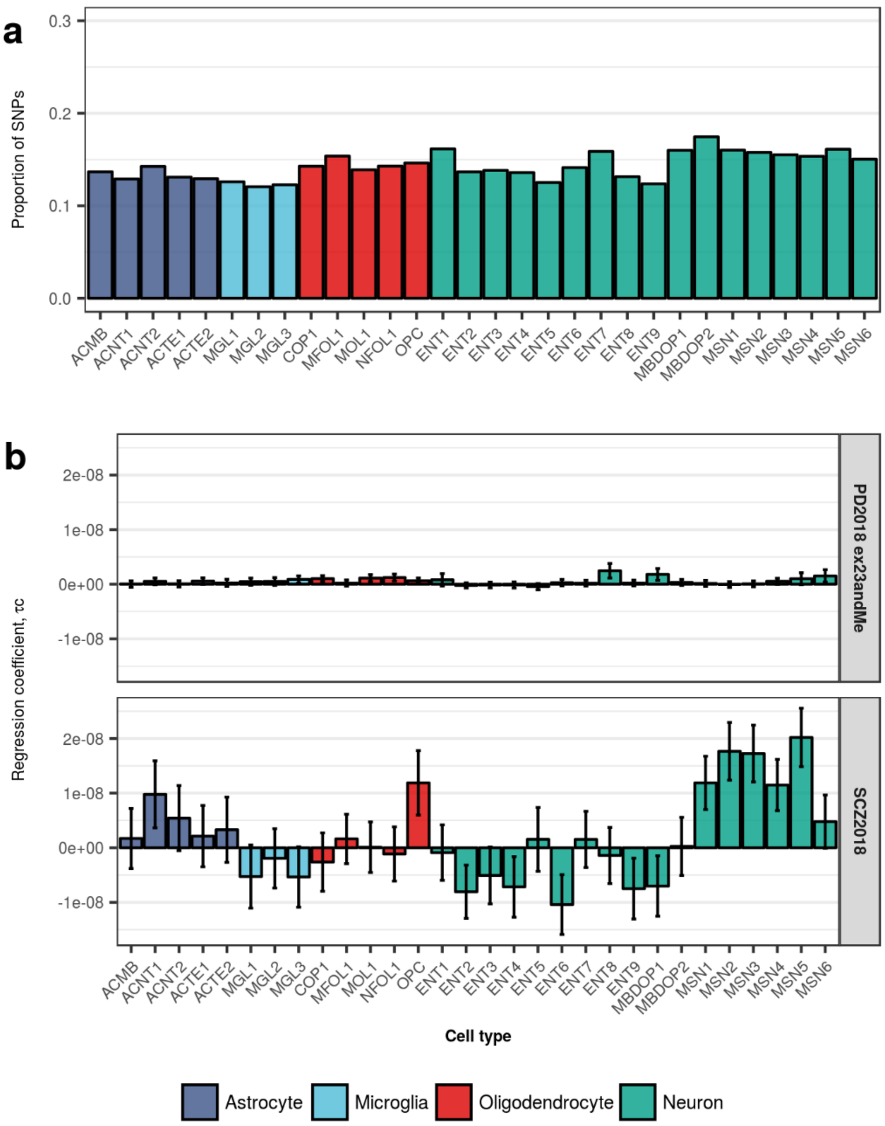

Supplementary Figure 10. SNP proportions (a) and regression coefficients (b) across scRNA-sequencing-based cell-type-specific gene expression annotations. SNP proportions (**a**) are in comparison to the baseline model, consisting of 9,997,231 SNPs. Cell types (**a, b**) were ordered alphabetically within each overarching cell type category. Error bars represent the standard error of the coefficient. This is outputted by the LDSC software, which estimates it using the covariance matrix for coefficient estimates.

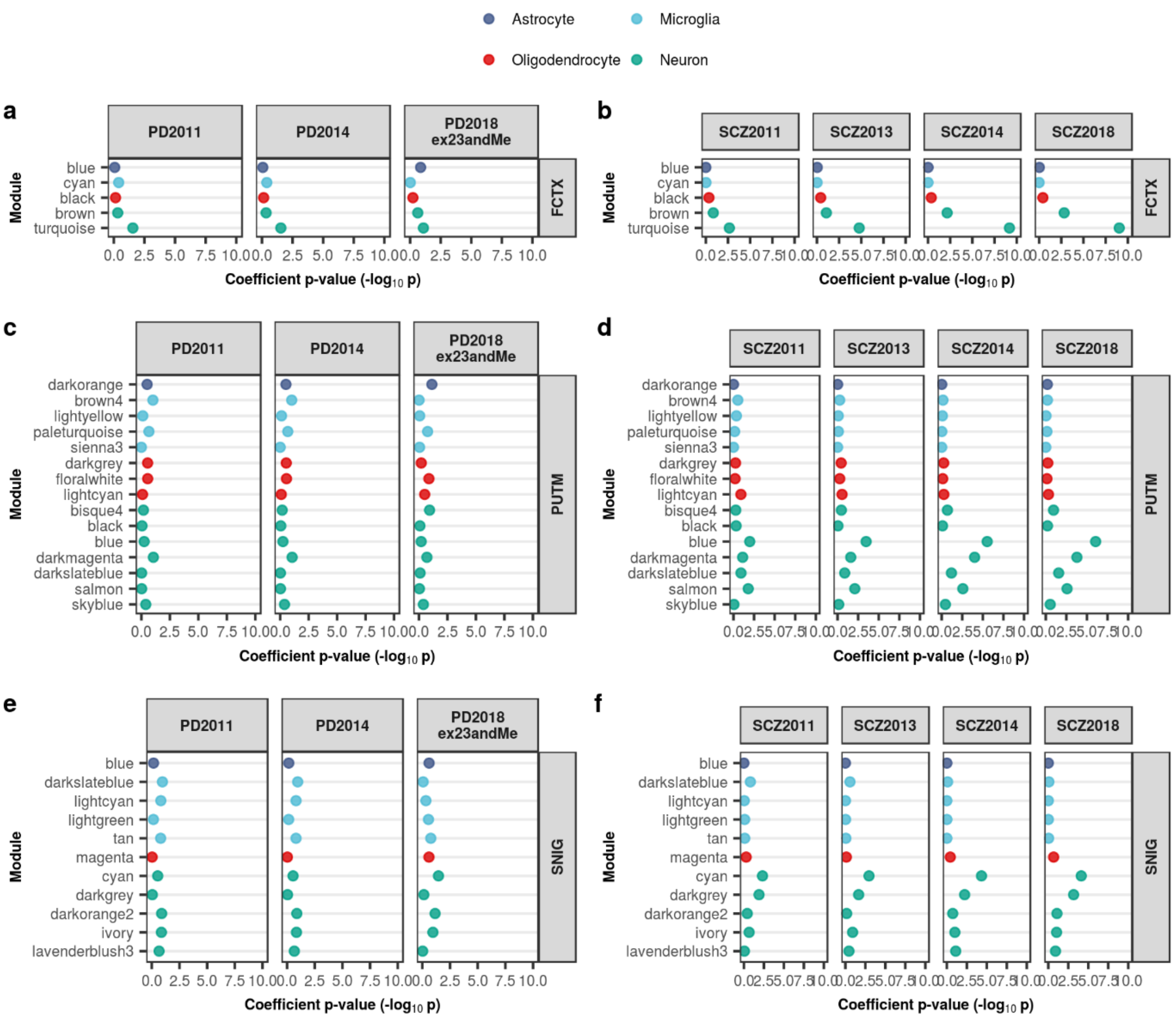

Supplementary Figure 11. Enrichment of PD and SCZ common-SNP heritability across GWAS iterations in cell-type modules inferred from human tissue-level co-expression networks.

Results for PD and SCZ in FCTX (**a, b**), PUTM (**c, d**) and SNIG (**e, f**). Cell-type-specific modules were ordered alphabetically within each overarching cell type category. Numerical results and module descriptions are reported in Supplementary Table 4. FCTX, frontal cortex; PUTM, putamen; SNIG, substantia nigra.

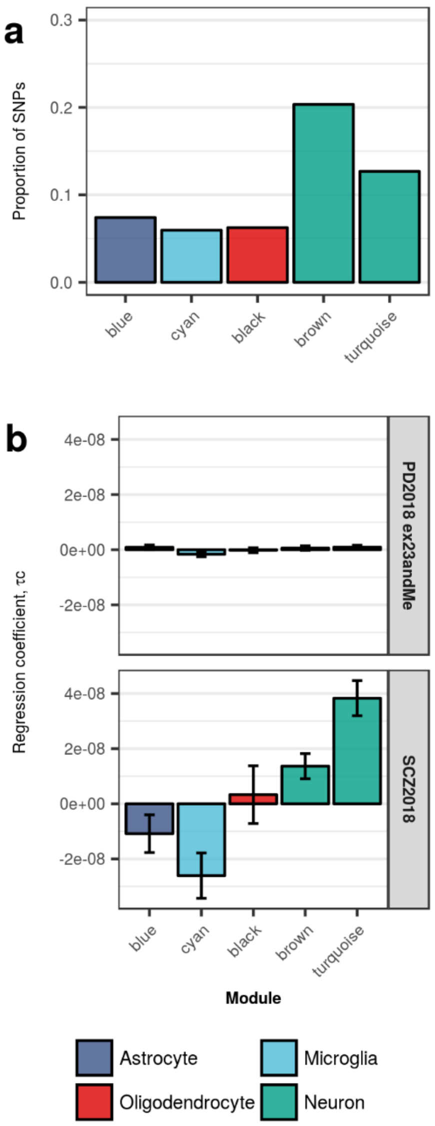

Supplementary Figure 12. SNP proportions (a) and regression coefficients (b) across frontal cortex-derived cell-type modules. SNP proportions (**a**) are in comparison to the baseline model, consisting of 9,997,231 SNPs. Modules (**a, b**) were ordered alphabetically within each overarching cell type category. Error bars represent the standard error of the coefficient. This is outputted by the LDSC software, which estimates it using the covariance matrix for coefficient estimates.

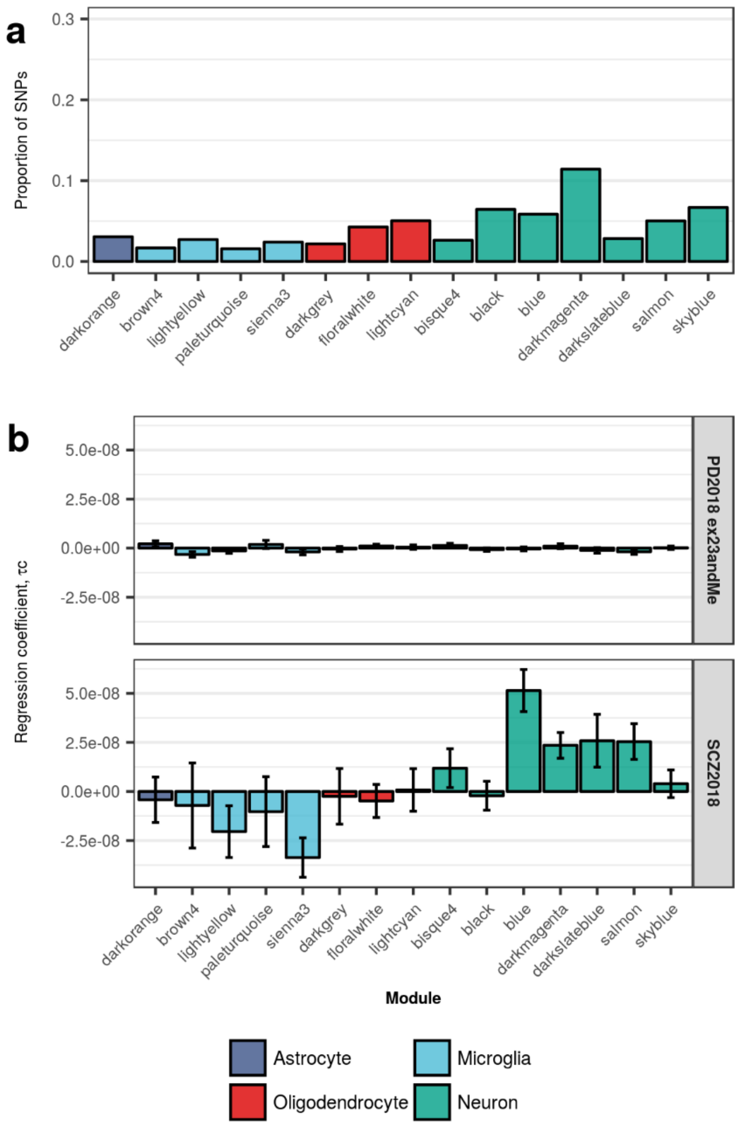

Supplementary Figure 13. SNP proportions (a) and regression coefficients (b) across putamen-derived cell-type modules. SNP proportions (**a**) are in comparison to the baseline model, consisting of 9,997,231 SNPs. Modules (**a, b**) were ordered alphabetically within each overarching cell type category. Error bars represent the standard error of the coefficient. This is outputted by the LDSC software, which estimates it using the covariance matrix for coefficient estimates.

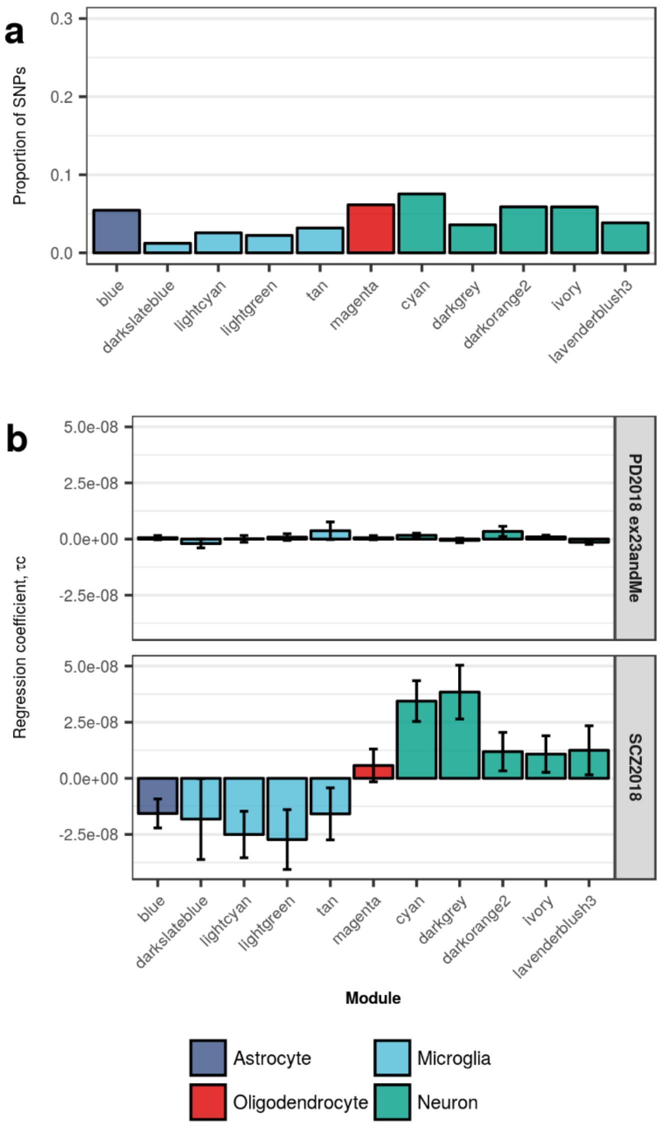

Supplementary Figure 14. SNP proportions (a) and regression coefficients (b) across substantia nigra-derived cell-type modules. SNP proportions (**a**) are in comparison to the baseline model, consisting of 9,997,231 SNPs. Modules (**a, b**) were ordered alphabetically within each overarching cell type category. Error bars represent the standard error of the coefficient. This is outputted by the LDSC software, which estimates it using the covariance matrix for coefficient estimates.

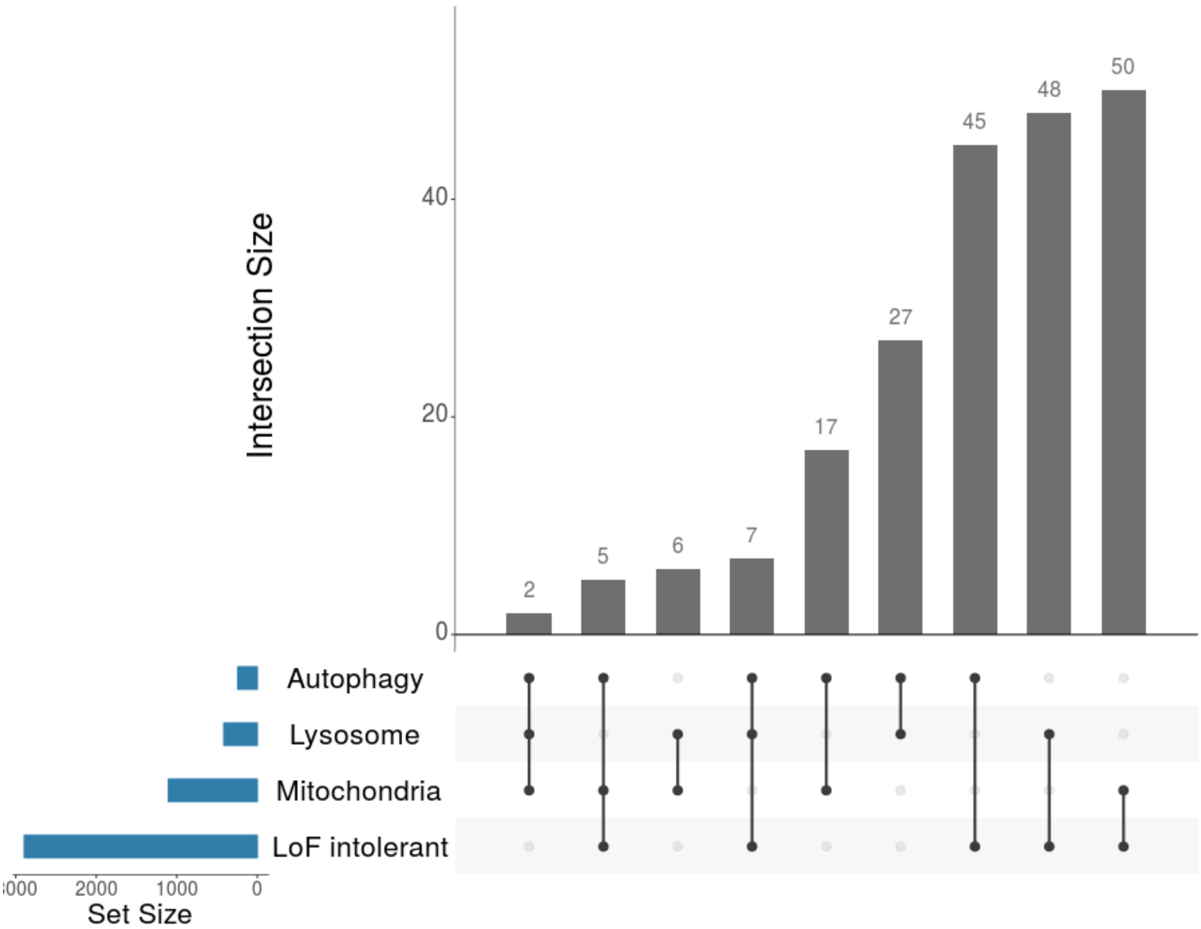

Supplementary Figure 15. Overlap between pathway gene sets.
Intersections were computed and visualised using Intervene (see URLs).

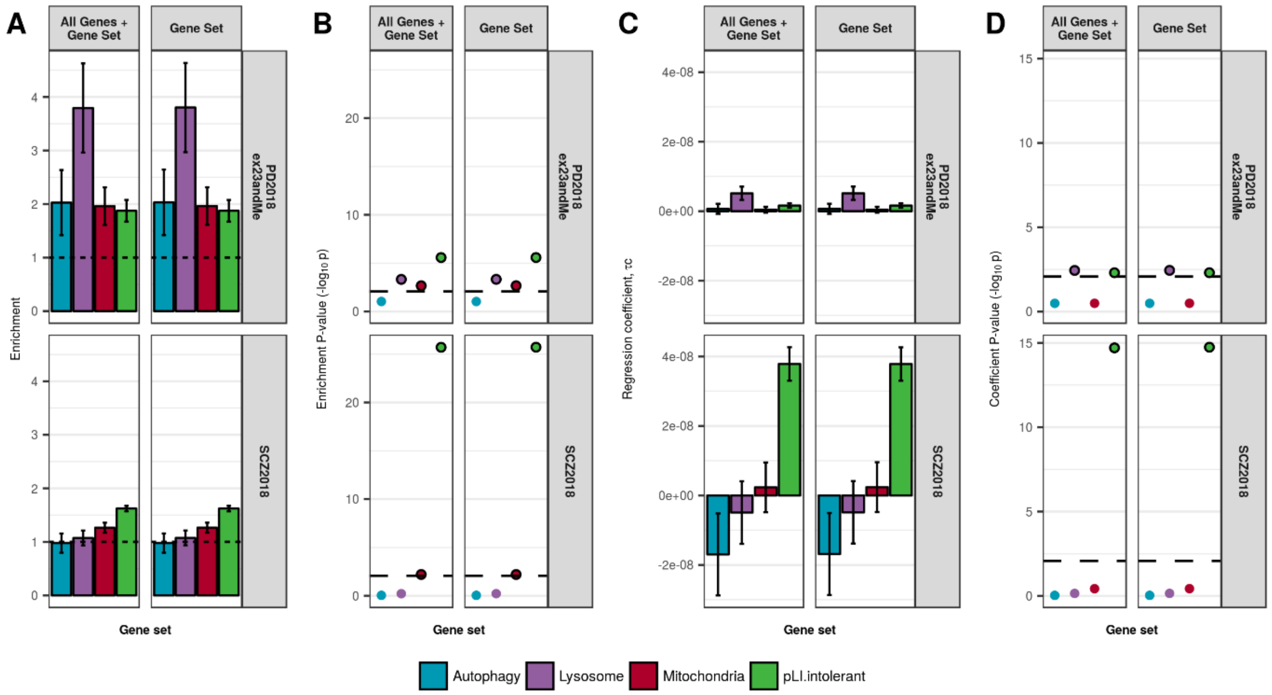

Supplementary Figure 16. Comparison of stratified LDSC estimates with and without "all genes" included in the regression. Results of stratified LDSC analyses using two different models: (1) baseline + all genes + gene set or (2) baseline + gene set. All outputs of LDSC are displayed, including (**a**) enrichment, which is equivalent to the proportion of heritability of an annotation divided by the proportion of SNPs attributed to that annotation; (**b**) the enrichment p-value, which does not account for the effect of including the baseline; (**c**) the regression co-efficient, a measure of whether an annotation category positively contributes to trait heritability conditional upon other categories included in the model; and (**d**) the co-efficient p-value. The black dashed line in (**a**) indicates no enrichment, while in (**b**) and (**d**) it indicates the cut-off for Bonferroni significance ($p < 0.05/(2 \times 4)$).

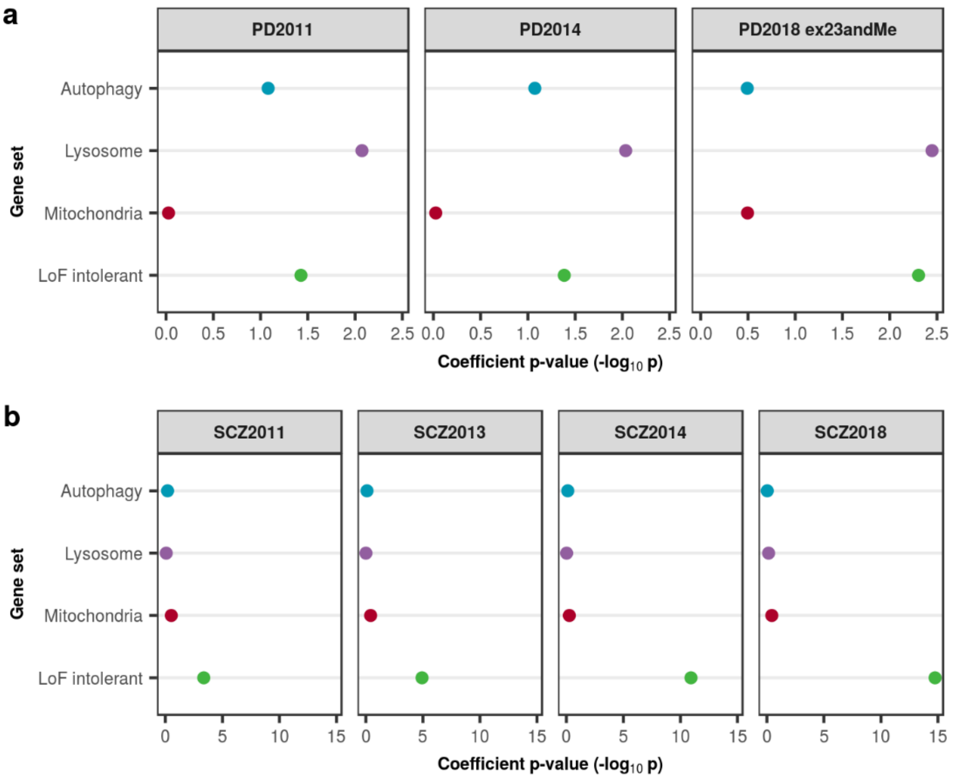

Supplementary Figure 17. Enrichment of PD and SCZ common-SNP heritability across GWAS iterations in gene sets.

(A) PD. (B) SCZ. Numerical results and module descriptions are reported in Supplementary Table 6.

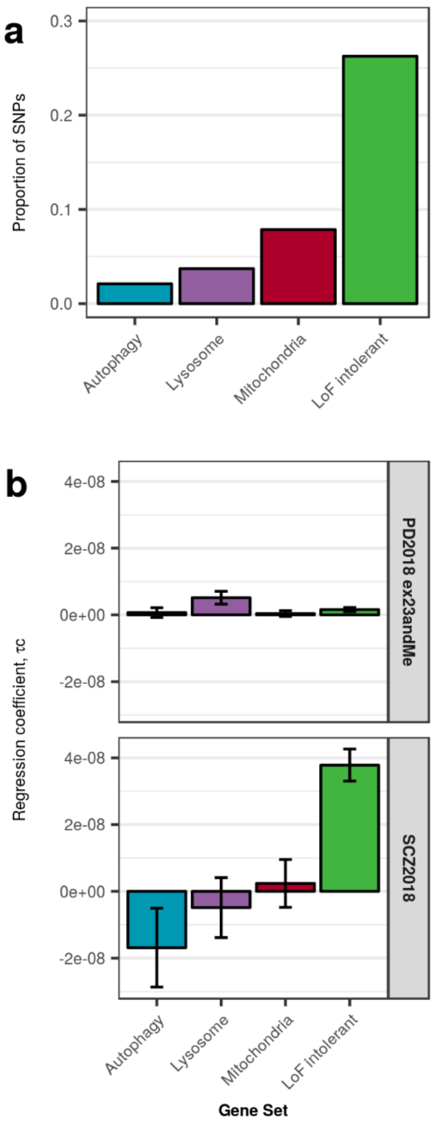

Supplementary Figure 18. SNP proportions (a) and regression coefficients (b) across gene sets. SNP proportions (**a**) are in comparison to the baseline model, consisting of 9,997,231 SNPs. Gene sets (**a, b**) were ordered alphabetically within each overarching cell type category. Error bars represent the standard error of the coefficient. This is outputted by the LDSC software, which estimates it using the covariance matrix for coefficient estimates.

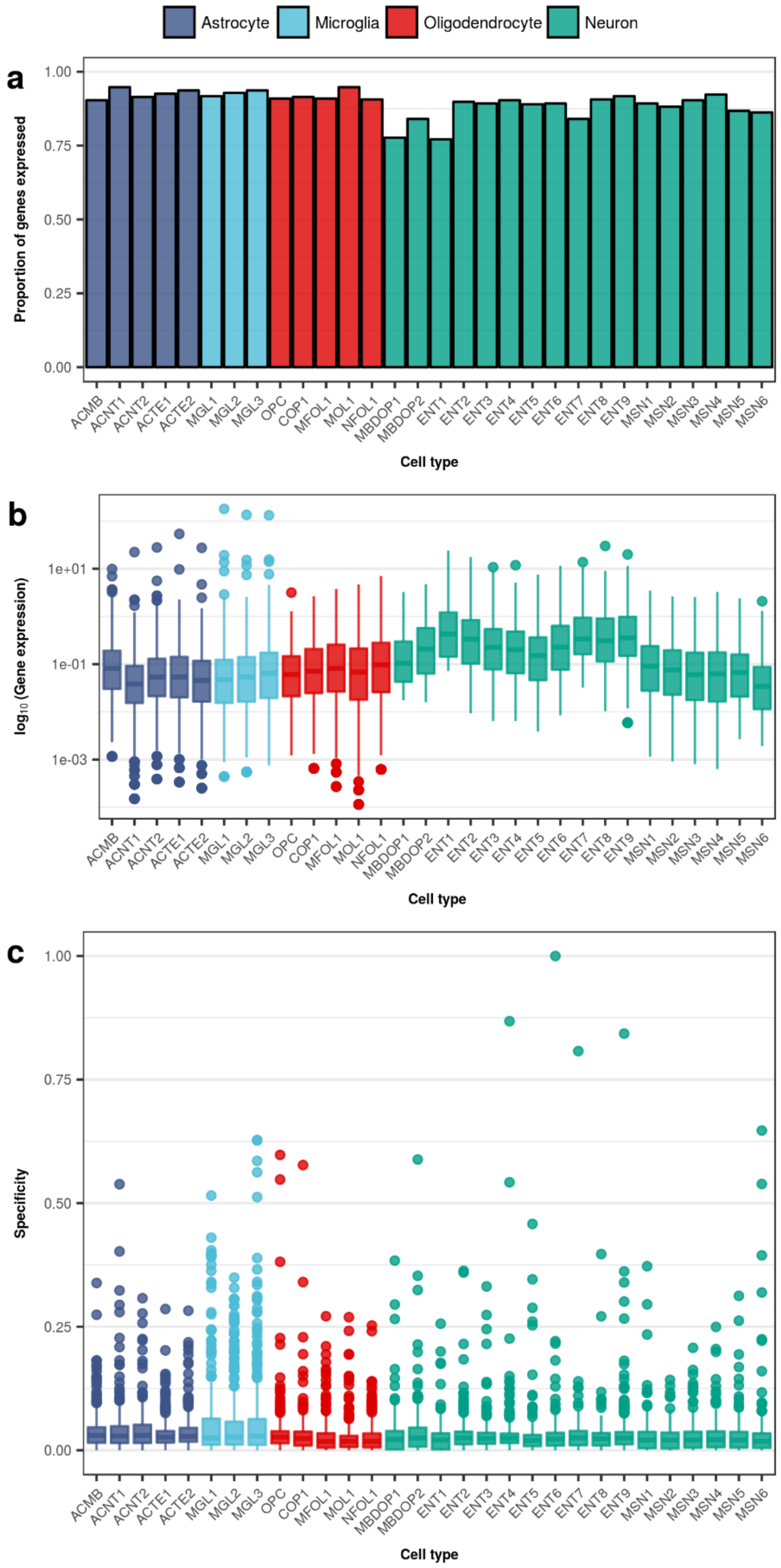

Supplementary Figure 19. The lysosomal gene set is ubiquitously expressed across all cell subtypes used in EWCE analyses. (**a**) Plotting the proportion of genes in the lysosomal gene set, with expression > 0, demonstrated that > 77% of the lysosomal gene set was expressed in all cell subtypes. Also plotted are gene expression values (**b,** plotted on a log_{10} scale due to some extreme expression values) and calculated specificity values (**c**), where 1 indicates a higher proportion of total expression in one cell type compared to all others.

Supplementary Tables

Supplementary Table 1.

Numerical results of stratified LDSC analyses across PD and SCZ GWAS iterations in tissue-specific gene expression annotations as used in Finucane et al. Spreadsheet 1: Column descriptions for all spreadsheets. Spreadsheet 2: Results relating to Figure 2A. Spreadsheet 3: Results relating to Figure 2B.

Supplementary Table 2.

Numerical results of stratified LDSC analyses across PD and SCZ GWAS iterations in tissue-specific eQTL annotations. Spreadsheet 1: Column descriptions for all spreadsheets. Spreadsheet 2: Results relating to Figure 3A. Spreadsheet 3: Results relating to Figure 3B.

Supplementary Table 3.

Numerical results of stratified LDSC analyses across PD and SCZ GWAS iterations in brain-related cell-type-specific gene expression annotations. Spreadsheet 1: Column descriptions for spreadsheets 4 and 5. Spreadsheet 2: Cell-type abbreviations. Spreadsheet 3: List of all mouse genes from the Linnarsson data with expression > 0 that were not found to have a human ortholog using Biomart. Spreadsheet 4: Results relating to Figure 4A. Spreadsheet 5: Results relating to Figure 4B.

Supplementary Table 4.

Numerical results of stratified LDSC analyses across PD and SCZ GWAS iterations in cell-type modules inferred from human tissue-level co-expression networks. Spreadsheet 1: Column descriptions for spreadsheet 3. Spreadsheet 2: Descriptions of all modules in Figure 5A-C, with enriched GO terms and cell-type markers. Spreadsheet 3: Preservation values for all GTEx modules in Figure 5A-C, compared to modules from co-expression networks constructed from UKBEC in frontal cortex, putamen and substantia nigra. Spreadsheet 4: Results relating to Figure 5A-C.

Supplementary Table 5.

Numerical results of MAGMA and EWCE analyses from Figure 6. Spreadsheet 1: All genes from the MAGMA analysis passing genome-wide significance ($p < 2.82 \times 10^{-6}$). Spreadsheet 2: Genes identified in ref.[42] as TWAS and colocalisation hits within dorsolateral prefrontal cortex tissue. Spreadsheet 3: Genes from the combination of the MAGMA and TWAS/coloc analyses, with overlapping genes removed. Spreadsheet 4: Bootstrapping results, as displayed in Figure 6.

Supplementary Table 6.

Numerical results of stratified LDSC and EWCE analyses from Figure 7. Spreadsheet 1: Gene lists used in stratified LDSC and EWCE analyses. Spreadsheet 2: Results of stratified LDSC analyses across PD and SCZ GWAS iterations. Spreadsheet 3: Bootstrapping results, as displayed in Figure 7B. Spreadsheet 4: Bootstrapping results, as displayed in Figure 7C.
